# Comparing patient characteristics and treatment processes in patients receiving physical therapy in the United States, Israel and the Netherlands: Cross sectional analyses of data from three clinical databases

**DOI:** 10.1186/1472-6963-8-163

**Published:** 2008-07-30

**Authors:** Ilse CS Swinkels, Dennis L Hart, Daniel Deutscher, Wil JH van den Bosch, Joost Dekker, Dinny H de Bakker, Cornelia HM van den Ende

**Affiliations:** 1 NIVEL; Netherlands Institute for Health Services Research; P.O. Box 1568; 3500 BN Utrecht, The Netherlands; 2 Focus On Therapeutic Outcomes, Inc., P.O. Box 114444, Knoxville, Tennessee 37939-1444, USA; 3 Maccabi Healthcare Services, 27 Hamered Street, Tel Aviv 68125, Israel; 4 Radboud University Medical Centre, Department of General Practice; P.O. Box 9101; 6500 HB Nijmegen, The Netherlands; 5 Department of Rehabilitation Medicine, EMGO Institute, VU University Medical Centre Amsterdam; P.O. Box 7057; 1007 MB Amsterdam, The Netherlands; 6 Sint Maartenskliniek, Department of rheumatology; P.O. Box 9011; 6500 GM Nijmegen, The Netherlands

## Abstract

**Background:**

Many assume that outcomes from physical therapy research in one country can be generalized to other countries. However, no well designed studies comparing outcomes among countries have been conducted. In this exploratory study, our goal was to compare patient demographics and treatment processes in outpatient physical therapy practice in the United States, Israel and the Netherlands.

**Methods:**

Cross-sectional data from three different clinical databases were examined. Data were selected for patients aged 18 years and older and started an episode of outpatient therapy between January 1^st ^2005 and December 31^st ^2005. Results are based on data from approximately 63,000 patients from the United States, 100,000 from Israel and 12,000 from the Netherlands.

**Results:**

Age, gender and the body part treated were similar in the three countries. Differences existed in episode duration of the health problem, with more patients with chronic complaints treated in the United States and Israel compared to the Netherlands. In the United States and Israel, physical agents and mechanical modalities were applied more often than in the Netherlands. The mean number of visits per treatment episode, adjusted for age, gender, and episode duration, varied from 8 in Israel to 11 in the United States and the Netherlands.

**Conclusion:**

The current study showed that clinical databases can be used for comparing patient demographic characteristics and for identifying similarities and differences among countries in physical therapy practice. However, terminology used to describe treatment processes and classify patients was different among databases. More standardisation is required to enable more detailed comparisons. Nevertheless the differences found in number of treatment visits per episode imply that one has to be careful to generalize outcomes from physical therapy research from one country to another.

## Background

Physical therapy services are essential components of health services delivery systems around the world, and physical therapy is one of the health care professions involved in the management of patients with limitations in physical functioning, which is a universal experience for all people. One aim of physical therapists is to identify and maximize human movement potential within the spheres of promotion, prevention, treatment and rehabilitation, in partnership with their patients [1]. In order to achieve scientific credibility and validate practice, research involving the practice of physical therapy has increased worldwide in the last decades [2-4]. Many people assume that evaluations of the number of visits per treatment episode, therapy duration and clinical outcomes from physical therapy studies in one country can be generalized to other countries. However, to date no well designed studies have been conducted comparing patient characteristics, treatment process characteristics, and outcomes in physical therapy among countries. According to the World Confederation of Physical Therapy, such comparisons are valuable for the development of the profession [5] as they allow countries to learn from each other.

International comparisons of physical therapy care can be performed by comparing data from clinical databases, i.e. collections of information from (electronic) medical records from many providers. In a previous study, our research team identified seven clinical databases in three different countries [6]. Data from these databases located in the United States, Israel and the Netherlands were used to initiate an international comparison of patient demographic characteristics and treatment process characteristics in outpatient physical therapy practice. The organization of physical therapy in these three countries is described below. To advance our research findings. we formulated the following research questions related to patients receiving outpatient physical therapy in the United States, Israel and the Netherlands:

- What were the patient demographic and health related characteristics?

- Which treatment processes were received?

- What were the relationships between the patient demographic and health related characteristics and the number of visits per episodes in the three datasets examined?

We formulated these questions for all patients in the databases. We replicated the analyses for patients with lumbar spine syndromes separately because, according to the data analyzed, patients with lumbar spine syndromes represent the most frequently treated group of patients in outpatient therapy in each of the three countries. Patients treated for lumbar spine impairments are a heterogeneous patient population [7]. Therefore, we decided to ask the questions as well for patients with ankle sprain, which we considered a more homogeneous patient population and an acceptable prevalence in all three databases.

### Descriptions of the organization of physical therapy in the USA, Israel and the Netherlands

#### USA

The health care delivery system in the United States is a mix of many different types of payers who are responsible for covering the expense of care delivery. For example, citizens or their employers commonly purchase health care benefits from private insurance companies that offer several different plans of coverage for the citizens/employees. Federal payers also exist that cover health care benefits to specific groups of people, like those individuals older than 65 years or who are impoverished. Employees of companies also are covered by state mandated workers' compensation plans in case the employee is injured while performing work-related tasks. In addition, people who do not want or cannot afford coverage from the private insurance companies, or their employers do not cover the cost of health care and the individual does not qualify for Federal programs, could pay for the health care benefits themselves. Therefore, the norm is a wide variety of plans of coverage for therapy services. Each plan has different rules governing coverage of physical therapy, so therapists or the companies for which they work must understand the rules in order for the successful billing of clinical services. Although there is increasing interest in value-based purchasing of health care in private insurance companies [8] and Federal programs [9] where clinicians would be reimbursed dependent on good outcomes delivered efficiently [10], few plans pay therapists on the value of the outcome provided for the therapy services. Currently, plans for clinical therapy services encourage higher delivery of procedures (because the therapy services are commonly reimbursed by procedural code) and volume of patients treated per time frame (because the amount of reimbursement has decreased by insurance companies in an attempt to control costs of services) [8,9]. Although some insurance plans will 'listen' to therapists if more or different than customary treatment is requested, there is no or limited incentive for therapists to provide evidence-based practice or to improve the outcomes of treatment. The percent of patients referred to therapy as evidenced in the FOTO database attests to the flexibility of the referral process to therapy. Although most states currently have state laws that allow therapists to practice without a referral from a physician, i.e., direct access, tradition and health care practices continue to encourage physician referral of patients to therapy. Therapists can be employed by large to small businesses, insurance companies, or practices privately held by therapists or physicians. Other business arrangements are possible. Most therapists are salaried, but some have bonus systems commonly based on productivity. Therapists were required to successfully complete an undergraduate program before becoming licensed to practice physical therapy, but most educational programs have changed or are changing to a post-graduate doctoral program, which will allow therapists to take the licensing examination.

#### Israel

Maccabi Healthcare Services is a public Health plan responsible for the healthcare of approximately 1.7 million people in Israel, which consists about 25% of the total population. In Israel, all citizens must be insured by one of the 4 public health plans, and payment is done by taxation relative to income. Health services coverage is defined by a general national health "basket" governmental law. However, some additional coverage may be chosen to be included in the basic health "basket" by each health plan. Referral to physical therapy is done by physicians and there is no direct access. All physical therapy is provided by over 400 employed therapists. Their salary is a fixed salary, except a bonus payment that is given within a range of average visits per hours. Additionally, each new patient is counted as two visits for the bonus payment. Although the national health coverage for PT has defined a maximum of 12 visits per episode of care per incident, the Maccabi PT management has decided that there would be no limit to the amount of episodes or number of visits per episodes for which a patient can be covered, as long as the therapists can provide clinical support for their decision to continue care. The fact that the average number of visits per episode of care is below the maximum number defined within the national health coverage, has facilitated this decision. Education of physical therapist includes a minimum of four years of academic education leading to a Bachelor in Physical Therapy.

#### The Netherlands

The Dutch health care system is a publicly funded health care system where general practitioners act as gatekeepers, controlling and coordinating access to specialty services. In the Dutch health care system in 2005, physical therapists were only accessible after referral by a physician. Over 90% of patients attending a physical therapist had been directly referred by their GP. The remaining 10% were referred by a medical specialist. People in the Netherlands had in 2005 either public or private health insurance, depending on their level of income. About 66% of the population was publicly insured. Public insurance cover for physical therapy was nationally regulated and in 2005 this meant that physical therapy was covered only when patients were suffering from a chronic condition, as specified on a list (about 12% of the patient population) and this coverage started at the tenth visit. People with public insurance were able to obtain additional private insurance that covered them also for the first nine visits and for physical therapy when they were not suffering from a chronic condition. Private insurance cover for physical therapy was not regulated at national level. The Dutch situation has changed in 2006. Currently, differentiation between public and private health care insurances has disappeared and physical therapy is accessible without a referral. In the Netherlands, 16% of the population contacted a physical therapist per year. Every physical therapy visit lasts about 25 minutes, and physical therapists are paid per visit, irrespective of the type of diagnosis and intervention. Nearly all therapists working in primary care are organised in private practices. Education of physical therapist consists of four years higher vocational education leading to a Bachelor in Health. In 2005, there were about 1,200 inhabitants per physical therapist.

## Methods

We analyzed data were used from three clinical databases: Focus On Therapeutic Outcomes, Inc. (USA), Maccabi Healthcare Services (Israel), and the National Information Service for Allied Health Care (the Netherlands). Data were selected for patients aged 18 years or older who started an episode of physical therapy care between January 1^st ^and December 31^st ^2005. This study was approved by the Institutional Review Board for the Protection of Human Subjects of FOTO and Maccabi. In the Netherlands, ethical approval was not obliged as patients were not subjected to treatment other than usual, nor were required to behave in a certain manner.

### Clinical databases

Focus On Therapeutic Outcomes, Inc. (FOTO) is a proprietary international medical-rehabilitation data management company from the United States that has been in existence since 1992 [6,11]. The FOTO network was developed for the purpose of generating an outcome-oriented, standardized information management system for use in outpatient physical therapy settings [12]. The company's purpose has been defined as: to provide reliable, valid and responsive outcomes measures and aggregate data management services to enable real-time information that empowers clinicians, patients, payers, and policy makers, and facilitates choice, delivery and payment based on the most effective rehabilitation therapy. In the current study, data of 1,004 physical therapists, working in 187 outpatient practices in 28 different states (U.S.) were used. More than 60% of outcomes data were entered via computer software employing computerized adaptive testing (CAT) methods [13-16], but paper and pencil data entry were available for clinics without computer availability. FOTO is the largest CAT generated outcomes data collection process for outpatient therapy in the world with over 2.4 million patient episodes and 700,000 CATs administered as of December 2007. Outcomes data are supplemented by process information and used by therapists to manage their patients in real time. Administrators use the data to manage the clinics and clinicians.

Maccabi Healthcare Services (Maccabi) is the second largest public Healthcare plan in Israel. Maccabi collects physical therapy data from over 70 outpatient clinics using several parallel informatics systems, which makes Maccabi the first health care service internationally to fully integrate electronic functional status outcomes assessment with an electronic medical record [17]: 1) electronic central medical file system; 2) electronic appointment management system; 3) central computer with the ability of querying the first two systems; and 4) computerized adaptive testing for functional outcomes measurement and data collecting. In the current study, data from 73 physical therapy clinics including over 400 therapists were used. Therapists use the outcomes, process and administrative data to manage their patients in real time, and both clinicians and physical therapy service managers use the data to improve patient management.

The National Information Service for Allied Health Care (LiPZ) is a computerized registration network in which about 100 Dutch physical therapists working in outpatient practices participate [6,18-20]. LiPZ was implemented in order to provide up-to-date information about the care provided by allied health care professionals in the Netherlands. LiPZ has been collecting health care related information since 2001. Participants use computer software to register their patients and treatments. In this software a special LiPZ-application is included, making it possible to register additional data and to make an export file every month. The data contain demographic information about patients visiting physical therapists, as well as information about the patient's condition and subsequent treatments. In the current study, data from 94 physical therapists, working in 43 practices were analyzed. LiPZ data are used for research purposes and administrators can use benchmark data to manage the clinics.

### Data set

None of the three data sets collected precisely the same information. However, there were similarities in data elements among all three databases, as the collection of data on patients' date of birth and gender, and on the profession of referring physicians. Furthermore, in all databases, the number of visits per episode, i.e. the number of times the patient had a face-to-face patient-therapist encounter, was collected. Data that needed recoding, because of differences between the datasets, were symptom episode duration, the patients' complaints and interventions. The recoding procedures are explained in the following paragraphs. These procedures were based on choices established on the basis of a consensus procedure among the authors.

Data on episode duration of the health problem, defined as the number of days between the date of onset of the condition and the date of therapy initial evaluation, was collected in all three networks as well, but the codes varied. In FOTO, the data were coded as '0 – 7 days', '8 – 14 days', '15 – 21 days', '22 – 90 days', '91 days – 6 months', '> 6 months'. In Maccabi, the data were coded as '0 – 21 days', '21 – 90 days', '> 90 days'. In LiPZ, the categories were '0 – 2 days', '3 – 7 days', '1 week – 1 month', '1 – 3 months', '3 – 6 months', '6 months – 1 year', '1 – 2 years', '> 2 years'. For our purposes, episode duration was recoded in 'acute' (less than 3 weeks in FOTO and Maccabi, less than 1 month in LiPZ), chronic (more than 3 months or 90 days) and sub acute (the category in between).

In all databases, information about the patients' complaints, e.g. reason for treatment, was collected. However, different classifications, with different levels of detail were used. As in all classifications the body part treated could be deducted, this was used as indication of the health problem of the patient. In FOTO, the patient, the front office staff or the therapist could select the body part treated. In Maccabi, the primary physical therapists' diagnoses were collected, using ICD-9 [21]. For the current study, these ICD-9 codes were recoded into the body part treated. In LiPZ, the reasons for referral as given by letter by the referring physician were coded by researchers using the International Classification of Primary Care (ICPC) [22]. These ICPC-codes were also recoded into the body part treated. Additional file 1 provides an overview of the response options in each database and the way they were summarized into the body part treated.

Interventions were collected in all databases, but time span of registration and classification differed. In FOTO, entry of interventions was optional for the therapist. When entered, each intervention is recorded for being applied at least once in the treatment episode or not at all. In Maccabi, the registration of intervention codes during the episode of care is mandatory, therefore the number of times each code was used during the overall episode of care is known. In LiPZ, at most three interventions applied in at least half of the treatment visits are registered at the end of the treatment episode. The different classifications are summarized into the following categories, deducted from the American Physical Therapy Association's (APTA) Guide to Physical Therapist Practice [23]: therapeutic exercises; functional training in work; manual therapy techniques; prescription, application, fabrication of devices; electrotherapeutic modalities; physical agents and mechanical modalities; and other. Additional file 2 gives for each database an overview of the response options and how they were summarized into the APTA categories.

The selection of patients with lumbar syndromes was based on the information about the reason for treatment, which was summarized into the body part treated as described above. The selection of patients with ankle sprain was based on the medical diagnoses, coded with ICD-9 in FOTO and Maccabi, and with ICPC in LiPZ, both using the same inclusion criteria.

### Statistical analyses

Descriptive statistics were calculated for the patient demographic and health characteristics and treatment processes characteristics. In the FOTO database there were over 25% missing cases for the profession of referring physicians variable. Therefore, for this variable the FOTO-data were not used. In all other variables and databases less than 25% missing cases were found. Differences in data were tested using χ^2^-tests for categorical variables and ANOVA for continuous variables. Differences in the number of treatment visits and treatment duration were tested using linear regression techniques controlling for gender, age and episode duration. To answer the questions about the number of visits and use of interventions, only data of patients for whom the treatment episode was closed were used.

For reasons of readability we used country names instead of database names in the results section.

## Results

### Patient demographic characteristics

There were subtle significant differences in gender and age of patients among the databases, but because the data sets are large and the differences were small from a practical sense, it appears that the demographic data are quite similar (Table 1). In the USA, more patients with lumbar spine syndromes tended to be female compared to patients in Israel or the Netherlands(p < 0.001). Patients in the USA or Israel tended to be older than patients in the Netherlands (p < 0.001). In patients with ankle sprain, similar differences were found for gender, but mean age of patients tended to be similar in all three databases (p = 0.391).

**Table 1:** Demographic characteristics of patients treated by a physical therapist in 2005 for the United States (FOTO), Israel (Maccabi) and the Netherlands (LiPZ), for the total patient population, patients treated for their lumbar spine and patients with ankle sprain

**Total population**		**FOTO**	**Maccabi**	**LiPZ**	**P**
Gender^1^	% male	37.2	39.6	42.0	<0.001
	% female	62.8	60.4	58.0	
					
Age^2^	% 18–44 years	33.8	35.0	40.7	<0.001
	% 45–64 years	42.7	39.3	37.9	
	% 65–74 years	13.7	15.0	11.1	
	% > 75 years	9.7	10.7	10.3	
					
	mean age (sd)	51.8 (16.2)	52.0 (17.0)	50.1 (17.2)	<0.001
	median age	52	52	49	
					
Number of patients		62,798	99,541	12,193	

**Lumbar spine treated**					

Gender^3^	% male	38.5	41.3	47.0	<0.001
	% female	61.5	58.7	53.0	
					
Age^4^	% 18–44 years	37.5	37.8	44.4	<0.001
	% 45–64 years	39.2	38.3	38.9	
	% 65–74 years	13.1	14.4	9.7	
	% > 75 years	10.2	9.5	7.0	
					
	mean age (sd)	51.0 (16.6)	51.1 (16.6)	48.7 (15.7)	<0.001
	median age	50	51	47	
					
Number of patients		18,878	22,166	2,057	

**Treated for ankle sprain**					

Gender	% male	40.3	43.8	52.9	0.022
	% female	59.7	56.2	47.1	
					
Age^5^	% 18–44 years	58.3	64.4	64.5	<0.001
	% 45–64 years	36.1	27.5	24.5	
	% 65–74 years	3.2	5.8	7.1	
	% > 75 years	2.4	2.3	3.9	
					
	mean age (sd)	41.5 (14.1)	40.4 (15.3)	40.6 (16.5)	0.391
	Median age	41	38	39	
					
Number of patients		472	1,463	155	

^1 ^Missing values FOTO n = 26, Maccabi = 0, LiPZ = 0

^2 ^Missing values FOTO n = 265, Maccabi = 0, LiPZ = 0

^3 ^Missing values FOTO n = 8, Maccabi = 1, LiPZ = 0

^4 ^Missing values FOTO n = 1, Maccabi = 0, LiPZ = 0

^5 ^Missing values FOTO n = 4, Maccabi = 0, LiPZ = 0

### Patient health characteristics

In the Netherlands, 38.0% of the patients had acute symptoms (< 1 month) (Table 2). In the USA and Israel, these percentages were lower, 18.4% and 14.3% respectively. A majority of the patients in the USA and Israel had chronic symptoms (> 3 months), while in the Netherlands, 35.2% of the patients had chronic symptoms. Similar results were found for patients treated for lumbar spine impairments. Compared to the total population, patients treated for ankle sprain more often had acute symptoms. But again, in the Netherlands this percentage was considerably higher than in the USA and Israel: 74.5%, 33.9% and 31.2% respectively.

**Table 2:** Percentage distribution of episode duration of health problem for patients treated by a physical therapist in 2005, for the United States (FOTO), Israel (Maccabi) and the Netherlands (LiPZ), for the total patient population, patients treated for their lumbar spine and patients with ankle sprain

**Total population**^1^	**FOTO**	**Maccabi**	**LiPZ**	**P**
Acute (0 – 21 days/1 month)^2^	18.4	14.3	38.0	<0.001
Sub acute (21 days/1 month – 3 months)^3^	28.4	31.2	26.7	
Chronic (>3 months)	53.2	54.5	35.2	
				
Number of patients (abs.)	62,713	84,523	10,793	

**Lumbar spine treated**^4^				

Acute (0 – 21 days/1 month)	20.4	11.2	49.9	<0.001
Sub acute (21 days/1 month – 3 months)^3^	24.4	25.5	23.9	
Chronic (>3 months)	55.2	63.3	26.1	
				
Number of patients (abs.)	18,873	19,809	1,950	

**Treated for ankle sprain**^5^				

Acute (0 – 21 days/1 month)	33.9	31.2	74.5	<0.001
Sub acute (21 days/1 month – 3 months)^3^	41.3	41.6	16.1	
Chronic (>3 months)	24.8	27.2	9.4	
				
Number of patients (abs.)	472	1,361	149	

^1 ^Missing values FOTO: 85; Maccabi: 15,016, LiPZ: 1,400

^2 ^Within FOTO and Maccabi: 0 to 21 days; within LiPZ 0 days to 1 month

^3 ^Within FOTO and Maccabi 21 days to 3 months; within LiPZ 1 to 3 months

^4 ^Missing values FOTO: 5; Maccabi: 2,357, LiPZ: 107

^5 ^Missing values FOTO: 0; Maccabi: 102, LiPZ: 6

In all three databases, the lumbar spine was the body part that was treated most frequently (Table 3), with percentages varying from 21.9% in the Netherlands to 30.6% in the USA. In all three networks, the neck, knee and shoulder are body parts that are treated frequently as well. In the Netherlands, over 55% of the patients were treated for spinal impairments. In the USA and Israel, this percentage was somewhat lower, 46.6% and 47.5% respectively.

**Table 3:** Percentage distribution of treated body part for patients treated by a physical therapist in 2005, for the United States (FOTO), Israel (Maccabi) and the Netherlands (LiPZ)*

	**FOTO**^1^	**Maccabi**^2^	**LiPZ**^3^
**Upper extremities**			
Shoulder	19.0	11.7	11.9
Arm (upper and/or forearm)	0.9	2.0	2.2
Elbow	1.4	3.0	2.8
Wrist/hand	1.4	7.4	1.7
*Total upper extremities*	*22.7*	*24.1*	*18.6*
			
**Lower extremities**			
Pelvis/hip	5.8	4.7	5.4
Leg (upper and/or lower)	3.1	1.5	4.9
Knee	14.3	14.5	11.5
Ankle/foot	7.4	7.8	3.0
*Total lower extremities*	*30.6*	*28.5*	*24.8*
			
**Spinal impairments**			
Craniofacial	0.2	0.2	1.5
Neck	13.6	18.2	20.0
Ribs/trunk	0.2	0.7	1.6
Thoracic spine	2.0	4.2	11.7
Lumbar spine	30.6	24.2	21.9
*Total spinal impairments*	*46.6*	*47.5*	*56.7*

Number of patients (abs.)	61,672	91,565	9,413
Unknown (abs.)	1,126	7,974	2,780

* Statistical analyses were not performed as the body parts were deducted from different classifications

^1 ^According to physical therapist, front office staff or patient

^2 ^Physical therapists' diagnosis recoded into treated body part

^3 ^Medical diagnosis recoded into treated body part

### Treatment process

The type of physicians referring patients to physical therapy differed between Israel and the Netherlands (Table 4). In Israel, 20.9% of the patients were referred by a general practitioner (GP), and about two third of the patients were referred by an orthopaedist. In the Netherlands, 89.9% of the patients were referred by a GP, while only 3.4% were referred by an orthopaedist. Differences were similar among all patient populations studied (p < 0.001). In the USA, patients in whom the type of referring physicians was known were mostly referred by a GP, an orthopaedist, a physiatrist, i.e. a physician specialised in physical medicine and rehabilitation, or an occupational medicine physician.

**Table 4:** Percentage distribution of profession of referring physicians for patients treated by a physical therapist in 2005, for Israel (Maccabi) and the Netherlands (LiPZ), for the total patient population, patients treated for their lumbar spine and patients with ankle sprain

**Total population**	**Maccabi**	**LiPZ**	**p**
GP	20.9	89.8	<0.001
Orthopaedist	67.4	3.8	
Neurologist	1.5	0.6	
Rheumatologist	0.4	0.2	
Other	9.9	5.6	
			
Number of patients (abs.)	99,541	12,193	

**Lumbar spine treated**			

GP	19.2	93.8	<0.001
Orthopaedist	73.3	0.4	
Neurologist	0.6	0.6	
Rheumatologist	0.2	0.0	
Other	6.7	5.1	
			
Number of patients (abs.)	22,166	2,057	

**Treated for ankle sprain**			

GP	8.7	95.5	<0.001
Orthopaedist	85.0	1.3	
Neurologist	0.0	0.0	
Rheumatologist	0.1	0.0	
Other	6.2	3.2	
			
Number of patients (abs.)	1,463	155	

In all three networks, therapeutic exercises were applied most frequently: in 78.0% of all patients in the USA, 79.4% of all patients in Israel and 84.5% of all patients in the Netherlands (Table 5). In the USA and Israel, physical agents or mechanical modalities were the second most frequently applied treatments (43.3% and 55.4% respectively), followed by manual therapy (31.8% and 54.7%, respectively). In the Netherlands, manual therapy was applied more often (67.2%), while physical agents or mechanical modalities were applied only in 5% of the patients. Results were comparable for patients with lumbar spinal impairments. In patients with ankle sprain, therapeutic exercises were applied more often compared to the total patient population. Furthermore, in the Netherlands, also the application of devices was a frequently applied treatment procedure, while in the USA and Israel physical agents or mechanical modalities and electrotherapeutic modalities and manual therapy were important treatment procedures.

**Table 5:** Percentage of interventions applied in patients treated by a physical therapist in 2005, for the United States (FOTO), Israel (Maccabi) and the Netherlands (LiPZ), for the total patient population, patients treated for their lumbar spine and patients with ankle sprain*

**Total population**	**FOTO**	**Maccabi**	**LiPZ**
Therapeutic exercise/advice	78.0	79.4	84.5
Manual therapy	31.8	54.7	67.2
Prescription, application and/or fabrication of devices	2.2	2.9	1.6
Electrotherapeutic modalities	22.5	41.7	4.5
Physical agents and mechanical modalities	43.3	55.4	5.0
Other	3.2	7.6	2.3
			
Number of patients – treatment closed (abs.)	36,076	96,568	8,869

**Lumbar spine treated**			

Therapeutic exercise/advice	86.0	81.2	82.5
Manual therapy	28.2	58.8	50.6
Prescription, application and/or fabrication of devices	1.2	1.2	0.1
Electrotherapeutic modalities	22.3	51.0	6.8
Physical agents and mechanical modalities	42.1	56.1	2.4
Other	4.6	5.0	1.4
			
Number of patients – treatment closed (abs.)	6,756	15,493	1,895

**Treated for ankle sprain**			

Therapeutic exercise/advice	89.0	91.9	87.8
Manual therapy	22.6	43.9	15.4
Prescription, application and/or fabrication of devices	4.9	4.5	41.5
Electrotherapeutic modalities	27.8	34.0	0.8
Physical agents and mechanical modalities	56.0	58.8	1.6
Other	6.1	2.6	2.4
			
Number of patients – treatment closed (abs.)	327	1,411	123

* because of the differences in classifications no statistical tests were conducted

Uncorrected mean numbers of visits per treatment episode in the total patient population were: 10.2 in the USA, 6.4 in Israel and 12.5 in the Netherlands. Corrected for age, gender and episode duration, mean numbers were 10.0 in the USA, 6.5 in Israel and 10.0 in the Netherlands. Patients with lumbar spine impairments from Israel had, corrected for age, gender and episode duration, on average 2.7 visits less than patients from the USA and on average 3.4 visits less than patients from the Netherlands. In patients with ankle sprain, differences in the corrected mean number of visits per treatment episode in the Netherlands and Israel were small (5.3 and 5.5, respectively). However, patients in the USA were treated more often (corrected mean number of treatment visits was 8.7).

Table 6 shows the effect of patient characteristics on the number of treatment visits in the three countries. Most regression coefficients are very small (less than 1, or in the case of age less than 0.10), meaning that the number of treatment visits deviates less than 1 (for age less than 0.1 treatment visit per year) from the reference group (male, 50 years, acute complaints). Only for patients with sub acute and chronic complaints we see considerably more treatment visits in the Netherlands (compared to the reference group 1.78 and 4.84 more respectively in the total population and somewhat less more for lumbar spine and ankle sprain). For patients in Israel the number of treatment visits varies hardly with patient characteristics. The USA takes an intermediate position with clearly more treatment visits for patients with chronic complaints treated for lumbar spine and ankle sprain but much less deviation from the reference group for the total population.

**Table 6:** Regression models for number of treatment visits in patients treated by a physical therapist in 2005, for the United States (FOTO), Israel (Maccabi) and the Netherlands (LiPZ), for the total patient population, patients treated for their lumbar spine and patients with ankle sprain

	FOTO			Maccabi			LiPZ		
**Total population**^1^	b	SE	p	b	SE	p	b	SE	p

Constant (male, 50 years, acute complaints)	9.95	0.099		6.52	0.043		9.98	0.279	
Female	-0.32	0.079	<0.001	0.32	0.039	<0.001	0.82	0.304	0.007
Age (in years)	0.01	0.002	<0.001	0.05	0.001	<0.001	0.13	0.009	<0.001
Sub acute complaints (reference = acute)	0.33	0.113	0.003	-0.01	0.052	0.953	1.78	0.378	<0.001
Chronic complaints (reference = acute)	0.60	0.103	<0.001	-0.80	0.046	<0.001	4.84	0.349	<0.001

**Lumbar spine treated**^2^									

Constant (male, 50 years, acute complaints)	8.09	0.143		5.44	0.102		8.85	0.409	
Female	-0.11	0.120	0.345	0.57	0.066	0.062	0.75	0.487	0.126
Age (in years)	0.003	0.002	0.173	0.04	0.002	0.158	0.05	0.015	0.003
Sub acute complaints	0.81	0.172	<0.001	0.06	0.115	0.005	1.57	0.607	0.010
Chronic complaints (reference = acute)	1.248	0.149	<0.001	-0.25	0.104	-0.026	2.25	0.583	<0.001

**Treated for ankle sprain**^3^									

Constant (male, 50 years, acute complaints)	8.74	0.666		5.48	0.302		5.26	0.681	
Female	-0.37	0.617	0.547	0.82	0.276	0.003	0.55	0.890	0.535
Age (in years)	0.01	0.021	0.759	0.03	0.009	0.001	0.01	0.027	0.597
Sub acute complaints	0.16	0.712	0.822	-0.35	0.307	0.251	1.56	1.181	0.188
Chronic complaints	2.59	0.783	0.001	0.64	0.340	0.058	3.09	1.394	0.028

^1 ^Number of patients FOTO n = 35, 129, Maccabi n = 96,562, LiPZ n = 10,348

^2 ^Number of patients FOTO n = 10,024, Maccabi n = 21,587, LiPZ n = 1,667

^3 ^Number of patients FOTO n = 324, Maccabi n = 1,411, LiPZ n = 146

## Discussion

The current study is the first to make comparisons of patient characteristics and treatment process characteristics in outpatient physical therapy practice in the United States, Israel and the Netherlands. These comparisons showed the data in three databases were remarkably similar in patient characteristics, like age, gender and body part treated. However, large differences were found in the episode duration of the health problems, the treatment procedures used and the number of treatment visits provided among countries.

### Patient demographic and patient health characteristics

The similarity in the body parts treated by physical therapists among countries implies that physical therapy practice is a definable area of clinical work. Apparently, the range of health problems is not highly influenced by the main sources of referring physicians, which did differ across the countries. However, large differences in the episode duration levels of the patients were found. The cause of this difference needs further investigation, but disparities in episode duration might be due to differences referral systems, with more patients referred by a general practitioner in the Netherlands than in the United States or Israel, or differences in waiting lists, which are short in the Netherlands and long in Israel, cultural factors, or use of other health professionals or medical agents. More rigorous designs would be needed to assess these differences. It would be interesting to include research into the consequences of these differences for the outcome of care in these designs as well.

### Treatment processes

Substantial differences were found in the interventions that were applied across the three countries. In general, Dutch physical therapists seem to have a more active approach and were more manual oriented, while in Israel and the United States, physical agents and mechanical modalities and electrotherapeutic modalities are frequently applied. Use of these agents and modalities has been decreasing since the 1990s in the Netherlands [18,24], which might be related to recommendations of the Health Council of the Netherlands which advised against the use of physical agents and modalities in many conditions [25].

Even within a homogeneous patient population, like patients with ankle sprain, large differences were found for interventions delivered among the countries studied. These disparities may be explained by differences in registration, i.e. the procedure of registration including the time span and the number of response options, and classification, i.e., the exact definitions of the response options. However, other explanations are possible as well. In patients with ankle sprain, considerable differences were found in the prescription, application and/or fabrication of devices, which may be explained by differences in the episode duration of the problems across countries. In the Netherlands, three-quarters of the patients with ankle sprain had acute complaints. In these patients, taping in combination with functional training appeared to be the favourable strategy when compared with immobilisation [26], what is also recommended in the Dutch clinical guidelines for the treatment of these patients [27]. In Israel and the United States, most patients had sub-acute or chronic complaints, in whom taping is only recommended as prevention for recurrent injury [28]. Therefore, it seems reasonable that devices are more often prescribed or applied in the Netherlands than in the United States or Israel. The higher use of physical agents and mechanical or electrotherapeutic modalities in the United States and Israel compared to the Netherlands, might be caused by cultural differences, but further research is needed into the exact reasons for these differences and into their effects on the outcome of care.

The mean number of visits per treatment episode differed among the three databases. In the total patient population and in patients treated for their lumbar spine, the number of visits per episode was higher in the United States and the Netherlands compared to Israel. In patients with ankle sprain, the number of treatment visits in the United States was higher than the number of treatment visits in Israel and the Netherlands. A remarkable finding is the narrow range of the mean number of treatment visits across different patient populations in Israel, while in the United States and especially in the Netherlands, the mean number of treatment visits differs extensively across patient populations. One explanation for the narrow range found in the population of Israel may be understaffing along with long waiting lists. Throughout the years, the Israeli database shows a continuous decrease in number of visits per episode of care along with a continuous increase in number of new patients per available clinical working hour. These circumstances narrow the flexibility of physical therapists in Israel and encourage fewer visits per episode, although it must be emphasised that from an administrative perspective, the Israeli system allows the therapist to provide any amount of visits per episodes as they feel is needed to achieve best possible outcomes. In comparison, patients receive more visits per episode in the United States and especially the Netherlands compared to Israel. We do not have the data nor were the data collected using a design that could elucidate why these differences in visits occurred, and therefore this finding awaits future research. Additionally, it is noteworthy that the direction of the regression parameters differed among the three databases. In the United States and in the Netherlands, patients with chronic complaints received more treatment visits than patients with acute complaints, while in Israel patients with chronic complaints received less treatment visits than patients with acute complaints. One hypothesis is understaffing in Israel resulting in long waiting lists might influence therapists' decisions to give less treatment visits to patients with chronic complaints as their predicted improvement is less than patients with more acute symptoms [10]. All these findings and hypothesis await sophisticated research designs, multivariate modelling and standardized operational definitions of all terms before the findings can be interpreted in a meaningful manner. Furthermore, research into the relation between the number of treatment visits and the outcome of care is needed in order to study the influence of treatment visits on outcomes.

## Limitations

This is the first study in which comparisons of patient characteristics and treatment process characteristics across different clinical databases of three countries have been made. Although this method has a number of advantages, it also has some limitations. First, the generalizability of our study is limited: we looked at only three countries and the representativeness of the three databases is debatable. Generalizability of the FOTO database is unknown, but the large size of the sample supports the potential that the data set could be representative of the type of patient treated in a typical outpatient therapy clinic in the United States. The Maccabi database was designed to examine physical therapy practice in one health maintenance organization (HMO) in Israel, and over 90% of patients covered by Maccabi Health Care System receiving therapy are included in the database. The Maccabi database probably comes closest to being representative of physical therapy practice in Israel because it is the second largest HMO in Israel and covers 25% of the population in Israel. The Dutch database is relatively small, but aimed at representativeness for the whole country. Practice and therapist characteristics of the LiPZ participants were compared to the characteristics of all Dutch physical therapists and practices as listed in a national register [29] showing no large differences on gender, age, practice size and urbanization rate [20]. Second, the databases differed in the variables included and when the same variables were assessed in each database, how the questions were asked and coded were different among the databases. This lack of standardization of data collection and operational definitions among the databases restricted the number of comparisons that could be made and eroded validity of the comparisons. In addition, it was not possible to compare outcomes among the databases because Israel used the same outcome measures as FOTO, but the Dutch outcome data were not comparable to the these measures. This lack of standardization of outcomes made it impossible to compare outcomes and the association between outcome and other process measures or patient demographic characteristics. Third, the reliability and validity of the medical and physical therapists' diagnoses and treatment procedures, which were not collected in a standardized way among the databases, are unknown. In an attempt to standardize terminology used in and related to physical therapy practice, APTA has written the Guide to Physical Therapists Practice [23]. This document includes an overview of physical therapy procedures. However, in the databases used for the current study, implementation of the APTA descriptions was not evident, although in the FOTO database therapists could enter APTA practice patterns. Nevertheless, we were able to use the language in the Guide to reorganize the classifications of treatments, so general comparisons could be made.

## Conclusion

The current study shows that clinical databases can be used for comparing similarities and differences in demographic and health related patient characteristics. However, for in-depth comparisons of diagnoses, interventions and outcomes, variables and measures need to be standardized among countries. Given the limitations in the databases and our comparisons, our results suggest that the number of treatment visits differs among the countries studied. If this finding can be confirmed, the finding has implications for the generalizability of physical therapy outcome research from one country to another.

## Recommendations

The authors would encourage international discussions on the desirability of standardizing and implementing operational definitions for data collected in these databases. As long no standardization takes place it is important to report in physical therapy outcome research on characteristics of patients and treatment processes in order to enable international readers to interprete the results in their own context. Finally, we advocate more international comparative research into physical therapy practice, both by involving more countries and by digging deeper in the causes of differences in for example number of treatment visits per episode.

## Competing interests

The authors declare that they have no competing interests.

## Authors' contributions

ICSS participated in the design of the study, performed the statistical analysis and drafted the manuscript. DLH, DD and CHMvdE participated in the design of the study and helped to draft the manuscript. WJHvdB helped to draft the manuscript. JD and DHdB contributed to interpretation of data and helped to draft the manuscript. All authors read and approved the final manuscript.

## Pre-publication history

The pre-publication history for this paper can be accessed here:



## Supplementary Material

Additional file 1 Recode reason for treatment. Overview of the recode procedure of the reason for treatment into bodypart treated. Click here for file

Additional file 2 Classification answer options applied interventions. Overview of the answer options for the applied interventions per database and the classification in which they were summarized. Click here for file
