# Supplementary material for: Comparing patient characteristics and treatment processes in patients receiving physical therapy in the United States, Israel and the Netherlands: Cross sectional analyses of data from three clinical databases
Source: BMC Health Serv Res. 2008 Jul 30;8:163. doi: 10.1186/1472-6963-8-163 (PMC2533658; doi:10.1186/1472-6963-8-163)
Supplement: Additional file 1 — Recode reason for treatment. Overview of the recode procedure of the reason for treatment into bodypart treated. [file 1472-6963-8-163-S1.doc]

**Appendix 1: Overview of the recode procedure of the reason for treatment into bodypart**

**treated**

|  | **United States (FOTO)** | **Israel (Maccabi):** | **The Netherlands (LiPZ):** |
| --- | --- | --- | --- |
| **Shoulder** | Shoulder | Above elbow amputation  Adhesive capsulitis shoulder  Biceps tendinopathy  Bursitis subacromial  Contusion shoulder/ upper arm  Dislocation shoulder closed  Erb’s Palsy  Fracture clavicle closed  Fracture of scapula  Impingement syndrome-shoulder  Infraspinatus tendinopathy  Muscle spasm/cramp/pain  Shoulder girdle/ arm  Myofascial pain syndrome – shoulder  girdle/arm  Pain shoulder  Rotator cuff tear  Surgery shoulder  Shoulder dislocation  Recurrent shoulder instability  Supraspinatus tendinitis-calcification  Teres Minor tendinopathy | Shoulder symptom/ complaint  Fracture clavicle  Shoulder dislocation  Shoulder syndrome |
| **Arm (upper and/ or forearm)** | Upper arm  Fore arm | Contusion upper limb  Fracture femur closed  Fracture femur base neck closed  Fracture of humerus  Reflex sympathetic dystrophy of   the upper limb  Fracture of radius  Fracture of radius and ulna  Fracture of ulna | Lymphedema armpit  Armpit symptom/ complaint  Arm symptom/ complaint  Fracture of humerus  Fracture of radius or ulna |
| **Elbow** | Elbow | Contusion elbow/ forearm  Elbow lateral epicondylitis (tennis elbow)  Elbow pain (joint)  Fracture elbow  Medial epicondylitis elbow  Muscle spasm/cramp/pain -  elbow/ forearm  Myofascial pain syndrome elbow/  forearm  Olecranon bursitis  Surgery elbow/ forearm  Ulnar nerve lesion – elbow | Elbow symptom/ complaint  Elbow lateral epicondylitis (tennis elbow)  Medial epicondylitis elbow |
| **Wrist/ hand** | Wrist  Hand | Contusion wrist/ hand  Fracture wrist  Muscle spasm/cramp/pain wrist/hand  Ulnar nerve lesion – wrist  Carpal tunnel syndrome  Contusion finger  De Quervain’s disease  Dupuytren’s contracture  Fracture carpal bones closed  Fracture hand bones multiple closed  Fracture metacarpals closed  Fracture phlalanges hand closed  Hand pain  Hand wound  Hand/ finger amputation  Mallet finger  Surgery hand/ wrist  Tendon repair hand  Trigger finger | Wrist symptom/ complaint  Hand/ finger symptom/ complaint  Fracture of phalanges hand  (Sub)luxation finger  Dupuytren’s contracture  Trigger finger  Carpal tunnel syndrome |
| **Pelvis/ hip** | Pelvis  Hip | Cystocele  Fracture of pelvis  Mixed incontinence  Pelvic (abdominal) pain - male  Pelvic (genital) pain - female  Pelvic fundus weakness  Uterine prolapse  Vulvodynia  Contusion hip/ thigh  Muscle spasm/cramp/pain - hip  Myofacial pain syndrome - hip  Pain hip  Revision of hip replacement  Hip hemiarthroplasty  Surgery hip  Total hip replacement  Tenosynovitis – hip  Trochanteric bursitis | Pelvic fracture  Stress incontinence  Urge incontinence  Urinary incontinence  Hip symptom/ complaint  Hip injury  Spina bifida occulta  Osteoarthrosis of hip |
| **Leg (upper and/ or lower)** | Upper leg  Lower leg without knee | Above knee amputation  Contusion lower limb  Fracture femur subcapital closed  IT band frinction syndrome  Refles sympathetic dystrophy of the lower limb  Achilles tendinitis  Fracture of fibula  Fracture of tibia  Fracture of tibia and fibula  Tendinitis tibialis ant or poste | Leg symptom/ complaint  Fracture femur  Injury achilles tendon  Fracture of tibia or fibula  Fasciitis plantaris |
| **Knee** | Knee | Below knee amputation  Bursitis infrapatellar/ tendonitis  Collateral ligament –disruption   lateral  Collateral ligament 0 disruption  medial  Contusion knee. Lower leg  Fracture of patella  Internal derangement knee  Knee arthroscopy  Knee menisectomy  Knee pain – local joint Knee – total knee replacement  Lateral meniscus tear knee  Lateral meniscus tear knee  current  Medial meniscus tear knee  Medial meniscus tear knee  current  Muscle spasm/cramp/pain  - knee  Myofacial pain syndrome knee  Osgood Schlatter’s disease  Patella tendinitis  Patellofemoral pain syndrome foot/  ankle kinetic dysfunction  Patellofemoral pain syndrome   hip/pelvic kinetic dysfunction  Patellofemoral pain syndrome local  joint  Patellofemoral pain syndrome  overuse  Pes-anserinus bursitis knee  Prepatellar bursitis/ tendinitis  Repair of the collateral ligaments  Repair of the cruciate ligaments  Revision of knee replacement  Surgical procedure knee  Sprain knee cruciate ligament  Tibial collateral ligament bursitis  Triad knee repair | Total knee replacement  Knee symptom/ complaint  Fracture patella  Sprain/ strain knee  Osteoarthrosis of knee  Acute internal damage knee  Chronic internal damage knee  Patellofemoral pain syndrome |
| **Ankle/ foot** | Ankle  Foot | Contusion ankle  Fracture of ankle  Muscle spasm/cramp/pain –  ankle/foot  Surgery ankle/ foot  Sprain ankle  Amputation foot  Amputation toe  Calcaneal spur  Clubfoot  Contusion foot  Contusion toe  Foot pain  Fracture phalanges foot closed  Fracture tarsal/ metatarsal bones  closed  Hammer toe acquired  Metatarsalgia  Plantar fasciitis | Ankle symptom/ complaint  Ankle distorsion  Foot/ toe symptom/ complaint  Fracture of phalanges foot  Pes planus |
| **Craniofacial** | Craniofacial | Dislocation jaw closed  Fracture of face bones  Head injury  Head injury – W/O mention of open  intracranial wound  Head injury with open intracranial  wound  Labyrinthectomy incision/ excision/  destruction of inner ear  Surgery jaw/ skull  Sprain jaw  TMJ disorder - articular disc dis  (reducing or nonreducing)  TMJ disorder – adhesions and  ankylosis (bony or fibrous)  TMJ disorder – lateral pterygoid  syndrome | Jaw symptom/ complaint  Headache |
| **Neck** | Neck | Cervical spine clinical instability  Cervicalgia  Cervicalgia with radicular syndrome  of upper limbs  Cervicobrachial syndrome  Contusion neck face or scalp  Muscle spasm/cramp/pain – cervical  Muofacial pain syndrome cervical  Surgery cervical  Spinal stenosis cervical  Tension headache  Torticollis  Whiplash injury | Neck symptom/ complaint  Whiplash injury  Syndromes cervical spine  Tension headache  Torticollis |
| **Ribs/ trunk** | Ribs/ trunk | Contusion trunk  Fracture ribs/sternum  Myofacial pain syndrome thorax  Surgery thorax  Sprain costo-vertebral joint | Chest symptom/ complaint  Flank/ axilla symptom/ complaint  Fracture rib  Tietze’s syndrome  COPD  Breast symptom/ complaint  Malignant neoplasm breast |
| **Thoracic spine** | Thoracic spine | Congenital postural deformities:  lordosis-scoliosis  Flat back  Fracture of vertebral column  Injuries spinal cord  Kyphosis acquired  Kyphosis adolescent postural  Lordosis acquired  Muscle spasm/cramp/pain - thoracic  Progressive infantile idiopathic  scoliosis  Round back  Scheurmann’s disease  osteochondrosis  Scoliosis (and kyphoscoliosis) -  idiopathic  Scoliosis acquired  Scoliosis adolescent idiopathic  Scoliosis cong  Scoliosis juvenile idiopathic  Scoliosis postural  Spinal stenosis thoracic  Thoracic spine clinical instability  Thoracic spine pain  Thoracogenic scoliosis | Position  Back symptom/ complaint  Fracture of vertebral column  Arthrosis/ spondylosis spine  Scoliosis  Lordosis  Kyphosis  Bulging disc |
| **Lumbar spine** | Lumbar spine | Coccyx pain  Compression lumbar nerve root  Low back pain  Lumbar spine clinical instability  Lumbosacral (or thoracic) pain with  radicular syndrome of lower limbs  Muscle spasm/cramp/pain - lumbar  Myofacial pain syndrome - lumbar  Laminectomy  Surgery lumbar  Sacroiliac pain  Sciatica  Spinal stenosis lumbar | Low back symptom/ complaint  Low back pain with radicular syndrome of lower limbs |
| **Unknown** |  | Acoustic neuroma excision  Administrative - physiotherapist  Aging  Amputation  Amyotrophic lateral sclerosis  Bell’s palsy (peripheral facial palsy)  Burn  Cerebral palsy  Chronic ulcer  Constipation  CVA  Dementia  Developmental coordination disorder  Dyspareunia  Edema  Epidemic vertigo  Falling  Fecal incontinence  Fibromyalgia  Guillain-Barre syndrome  Juvenile osteochondrosis  Labyrinthine dysfunction  Labyrinthine fistula  Labyrinthitis  Lipedema  Lymphedema  Lymphedema cong  Lymphedema due to abdominal  surgery  Lymphedema due to burns  Lymphedema duet o orthopedic  surgery  Lymphedema due to trauma  Lymphedema due to vascular disease  Median nerve neuropathy  Meniere’s disease  Meniere’s disease active   cochleovestibular  Multiple fractures  Multiple Sclerosis  Neuropathy peripheral  Parkinson’s disease  Poliomyelitis acute  Polyneuropathy sec to diabetes  mellitus  Postlumpectomy lymphedema  Postmastectomy lymphedema  syndrome  Respiratory system disfunction  Rheumatoid arthritis  Temporomandibular joint pain –  dysfunction syndrome  Thoracic outlet syndrome  TIA – non-specified  TIA – vertebrobasilar artery  Trigeminal neuralgia  Trigger point - muscular  Ulnar nerve lesion  Vaginismus organic  Vaginitis/ vulvovaginitis  Vertigo benign paroxysmal positional vertigo due to ototoxicity  Vestibular neuronitis  Vesitibulitis (gynecology)  Walking difficulty | Pain general  Weakness/ tiredness general  Swelling  Limited function/ disability  Infectious disease  Malignancy  Trauma/ injury  Multiple trauma  Adverse effect medical agent  Complication of medical treatment  Effect prosthetic device  Congenital anomaly  No disease  General disease  Blood symptom/ complaint  Hodgkin’s disease/ lymphoma  Leukaemia  Malignant neoplasm blood other  Blood/ lymph/spleen disease other  Abdominal pain/ cramps  Rectal/ anal pain  Incontinence of bowel  Swallowing problem  Digestive symptom/complaint other  Malignant neoplasm digest other  Eyelid symptom/ complaint  Tinnitus, ringing/buzzing ear  Vertiginous syndrome  Swollen ankles/ oedema  Limited function/ disability -  cardiovascular  Cardiovascular symptom/ complaint  Ischaemic heart disease w. angina  Acute myocardial infarction  Ischaemic heart disease w/o angina  Heart failure  Heart valve disease  Heart disease other  Hypertension uncomplicated  Transient cerebral ischaemia  Stroke/Cerebrovascular accident  Atherosclerosis  Varicose veins of leg  Haemorrhoids  Cardiovascular disease other  Muscle pain  Fibromyalgia  Muscle symptom/ complaint  Joint symptom/ complaint  Symptom/ complaint  musculoskeletal  Infections musculoskeletal system  Malignant neoplasm musculoskeletal  Fracture hand or foot  Fracture other  Sprain/ strain joint  Dislocation/ subluxation  Injury musculoskeletal  Congenital anomaly musculoskeletal  Synovitis  Rheumatoid arthritis  Bechterew’s disease  Osteoartrhosis other  Osteochondrosis  Osteoporosis  Acquired deformity of limb  Musculoskeletal disease  Tingling fingers/ feet/ toes  Sensation disturbance  Vertigo/ dizziness  Paralysis/ weakness  Limited function/ disability - neurological  Neurological symptom/ complaint  Malignant neoplasm nervous system  Concussion  Head injury other  Injury nervous system other  Congenital anomaly neurological  Multiple sclerosis  Parkinson’s disease  Migraine  Facial paralysis/ bell’s palsy  Peripheral neuritis  Thoracic outlet syndrome  Guillain-Barre syndrome  Neurological disease other  Feeling anxious/ nervous/ tense  Dementia  Anxiety disorder/ state  Somatization disorder  Neuraesthenia/ surmenage  Phobia/ compulsive disorder  Shortness of breath  Breathing problem  Sputum/ phlegm abnormal  Limited function/ disability - respiratory  Upper respiratory infection acute  Acute bronchitis  Pneumonia  Respiratory infection other  Malignant neoplasm bronchus/ lung  Asthma  Hyperventilation syndrome  Respiratory disease other  Pain/ tenderness of skin  Skin symptom/ complaint  Herpes zoster  Moniliasis/ candidiasis skin  Skin infection other  Malignant neoplasm of skin  Psoriasis  Chronic ulcer skin  Skin disease  Obesity  Diabetes Mellitus  Endocrine/ metabole/ nutrition  disease  Urination problems other  Bladder symptom/ complaint  Cystitis/ urinary infection other  Malignant neoplasm of kidney  Urinary disease other  Pregnancy symptom/ complaint  Uterovaginal prolapse  Genital disease female  Malignant neoplasm prostate |
