# Supplementary material for: Comparing patient characteristics and treatment processes in patients receiving physical therapy in the United States, Israel and the Netherlands: Cross sectional analyses of data from three clinical databases
Source: BMC Health Serv Res. 2008 Jul 30;8:163. doi: 10.1186/1472-6963-8-163 (PMC2533658; doi:10.1186/1472-6963-8-163)
Supplement: Additional file 2 — Classification answer options applied interventions. Overview of the answer options for the applied interventions per database and the classification in which they were summarized. [file 1472-6963-8-163-S2.doc]

**Overview of the answer options for the applied interventions per database and the classification in which they were summarized**

|  | **United States (FOTO):** | **Israel (Maccabi):** | **The Netherlands (LiPZ):** |
| --- | --- | --- | --- |
| **Therapeutic exercise** | ADL training  Aquatic exercises  Balance  Biofeedback/ EMG/  Body mechanics training  Closed chain exercises  Community reintegration  Cranio-sacral techniques  Developmental  Endurance  Energy conservation  Flexibility  Functional training  Gait training  Home exercises  Home management/self care  Incoordination, dexterity retraining  Joint mobility  McKenzie program  MET exercise (metabolic equivalent)  Muscle energy  Oral function/feed  Oromotor exercises  Pain modulation  Pelvic floor exercises  Perceptual exercises  Plyometrics  Postural exercises  Proprioceptive exercises  Prostetic training  Relaxation techniques  Running/ agility drills  Sensory re-education  Speech/communication  Stabilization  Strength  Stretching  Swallowing exercises  Swiss ball exercises  Therapeutic activity  Video-feedback training  Visual-motor retraining  Other exercises | ADL training  Aerobic exercise  Back school  Bed transfers exercise  Biofeedback/ EMG  Education for self exercise  Functional exercise  Gait exercise  Group exercise  Individual exercise at clinic  McKenzie program  MET exercise (metabolic equivalent)  Muscle energy  Physical exercise consultation and planning  PNF (proprioceptive neuromuscular facilitation)  Proprioceptive exercises  SET-therapy master (sling-based exercise)  Sitting active exercise  Sitting assisted active exercise  Sitting balance reaction exercise  Sitting passive exercise  Stabilization exercise  Standing balance reaction exercise  Stationary bike exercise  Strengthening exercise  Stretching exercise  Supine active exercise  Supine assisted active exercise  Supine passive exercise  Transfer exercise  Treadmill exercise | Exercise therapy – training of activities  Exercise therapy – training of functions  Information/ advice |
| **Manual therapy** | Augmented soft tissue massage Augmented soft tissue mobilization  Friction massage  Manipulation (high velocity)  Massage  Mobilization  Myofascial technique  Traction | Deep friction massage  Hold/ relax  Joint mobilization  Neural tension/ mobilization  Segmental manipulation  Soft tissue mobilization  Strain-counterstrain | Manual massage  Manual manipulation |
| **Prescription, application, fabrication of devices** | Casting  Dynamic splints  Orthotics/splinting/bracing  Prosthetic modification  Therapeutic taping | Home adaption of devices/ wheelchair  Taping | Fabrication of devices  Application of devices  Testing of devices |
| **Electrotherapeutic modalities** | Electrical stimulation/pain  Electrical stimulation/retraining  Electrical stimulation/strength  Iontophoresis | Electrotherapeutic modalities for iontophoresis management  Electrotherapeutic modalities for oedema management  Electrotherapeutic modalities for pain management  Motor TENS  Short wave | Electrical stimulation |
| **Physical agents and mechanical modalities** | Contrast baths  CPM (continuous passive motion)  Cryocuff/compression  Diathermy  Ice/cryotherapy  Moiste heat  Phonophoresis  Ultrasound  Whirlpool  Other agents | Cervical mechanical traction  Cold pack  CPM (continuous passive motion)  Cryocuff/compression  Hot pack  Lumbar mechanical traction  Lymph press  Paraffin bath  Ultrasound  Whirlpool | Mechanical modalities  Thermical energy Electromagnetic (UKG, laser) |
| **Other** | Cognitive linguistic function  Cognitive retraining  Speech/communication  Aural rehab  Wound care  Work simulation  Work hardening tasks  Other procedures | Administrative processes | Instrumental interventions  Medication  Other |
